# Conscious Exploration of Alpha-Cuts in the Parametric Solution of the School Bus Routing Problem with Fuzzy Walking Distance

**DOI:** 10.1155/2022/4821927

**Published:** 2022-06-08

**Authors:** Eduardo Sánchez-Ansola, Ana C. Pérez-Pérez, Alejandro Rosete, Isis Torres-Pérez, Omar Rojas, Guillermo Sosa-Gómez

**Affiliations:** ^1^Facultad de Ingeniería Informática, Universidad Tecnológica de La Habana José Antonio Echeverría (Cujae), Marianao 19390, La Habana, Cuba; ^2^Facultad de Ciencias Económicas y Empresariales, Universidad Panamericana, Álvaro del Portillo 49, Zapopan 45010, Jalisco, Mexico; ^3^Faculty of Economics and Business, Universitas Airlangga, Surabaya, Indonesia

## Abstract

Combinatorial optimization problems allow for modeling multiple situations in which proper allocation of resources is needed. For some real-world problems, the use of fuzzy elements in the models allows for incorporating certain levels of uncertainty to better approximate such real-world situations. One way to solve combinatorial optimization problems with fuzzy elements is the parametric approach, where it is necessary to define how to explore different relaxation levels using alpha-cuts. Researchers tend to select such alpha-cuts uniformly. The current investigation proposes a novel strategy for selecting alpha-cuts in the School Bus Routing Problem with fuzzy students' maximum walking distance. This proposal bases its foundations on the number of student-bus stop pairs available according to the different levels of relaxations allowed. Results demonstrate how the proposed strategy gives attractive solutions with more diverse trade-offs, contrasted with other methods in the literature. Furthermore, it decreases the computational cost for those instances where the maximum relaxation does not provide new pairs of students-bus stops.

## 1. Introduction

Decreasing environmental pollution, reaching responsible consumption, and setting up access to transportation are fundamental objectives for human society to achieve sustainable development, according to the 2030 United Nations' agenda for sustainable development [1]. The proper use of science and technology might contribute to the fulfillment of such objectives, and in particular, Artificial Intelligence (AI) and soft computing (SC) are excellent candidates for such an endeavor [2, 3]. One of the areas where AI has had a greater impact is modeling and solving optimization problems based on transportation planning [4].

One of the current challenges in modeling optimization problems is to make them closer to contemporary reality's requirements [5]. In this way, the use of fuzzy optimization can be a valuable approach because, in many cases, there is a total or partial absence of information, knowledge, understanding, or awareness of a potential event's occurrence. Thus, certain levels of uncertainty need to be introduced [6]. Numerous authors have employed fuzzy elements to model optimization problems [7–11]. In particular, there are several engaging solutions to transportation planning problems, like the numerous variants of the Vehicle Routing Problem (VRP) [12–14].

A proven way to reach good results in the resolution of fuzzy optimization problems is through the parametric approach [15]. By this approach, a fuzzy problem is transformed into a set of crisp instances of the problem by defining a set of alpha-cuts. The union of the solutions of each of these instances forms the final solution of the original fuzzy problem. Usually, the authors define the alpha-cuts based on experience or somehow arbitrarily. The authors of [16] presented a method to determine the alpha-cuts in a better way, using an adaptive approach similar to a binary search technique. The results showed the advantages of their proposal to give a more meaningful set of solutions to the decision-maker.

The School Bus Routing Problem (SBRP) is one of the up-to-date transportation optimization problems, as seen in [5]. This problem aims to create an optimal set of routes to transport the students from/to a set of selected bus stops to/from their school [9]. Many authors, like [17–19], guide their research on the SBRP for practical applications that lead to significant cost savings for those affected by such applications.

In [20], a model for the SBRP with Fuzzy Walking Distance (FWDSBRP) was presented, and the parametric approach was used in its solution. In this case, the explored alpha-cuts were arbitrarily selected (uniformly distributed in the interval [0, 1]) to explore the space of fuzzy solutions. This arbitrary selection of the alpha-cuts can bring with it some difficulties: the unnecessary exploration of all the alpha-cuts even though there are no differences in the quality of the solution obtained with each one of them; little or no variation between the quality of the set of solutions in terms of the objective function. It is worth noting that the variation of the relaxation (in terms of alpha values) is not necessarily proportional to the variation of the value obtained in the objective function. Therefore, it is important for the decision-maker to have a set of solutions with diversity in terms of the objective function based on more intelligent and conscious relaxations.

The main goal of this paper is to present a wiser strategy to explore the alpha-cuts, based on previous knowledge of each instance of a School Bus Routing Problem with Fuzzy Walking Distance (FWDSBRP). In particular, the proposed strategy uses information about the distances between students and bus stops to guide the exploration. Experimental results show that better results can be obtained with such a strategy in the sense of less redundant solutions and more diverse quality trade-off values for decision-makers.

The rest of the document is organized as follows. Materials and Methods address the general characteristics of the SBRP, the mathematical model and the fuzzy approach for the SBRP, and the description of the proposed strategy. Results and Discussion presents and discusses the experimental results with the proposed alpha-cuts selection strategy, and a comparison is conducted between the proposal and other alpha-cuts selection strategies. Finally, the conclusions and future work are presented.

## 2. Materials and Methods

### 2.1. School Bus Routing Problem

The SBRP is a combinatorial optimization problem with a specific type of VRP [21]. The main goal of this problem is to reach a cost-effective set of bus routes to transport the appropriate students from a set of designated bus stops to their school [22]. An adequate solution to the problem can only be reached when various constraints, such as the bus capacity, the maximum time that students can travel on the bus, or others, are satisfied [5]. It is important to note that despite the model being commonly expressed in terms of schools and students, it may be relevant too for other institutions.

As stated by [5], SBRP is a complex problem that can be divided into some less-complex subproblems. These subproblems are the selection of bus stops, the generation and scheduling of the routes, the adjustment to the school bell time, and the strategic transportation policy. As observed in [5], many authors focus their research on one subproblem or a mix of two or more of them. For instance, the authors of [23] presented an SBRP with multiple schools that include three of the previous subproblems. Meanwhile, [24] only focuses on the subproblem of the construction of the routes. From the point of view of the objectives, some authors focus their efforts on solving the SBRP with a single objective [18, 25, 26], and others, like [27, 28], attempt to solve the SBRP with multiple objectives.

More recently, researchers have focused their studies of SBRP on more realistic situations. An example of that can be seen in [17], where the main contributions are to assume gender separation and the special needs of some students. In [29], they considered the possibility of demand outsourcing (e.g., students using parallel systems for transportation). Finally, [30] introduced an SBRP with fuzzy constraints related to the capacity of the buses and the maximum distance that each student can walk, and also the consideration of special students' needs.

### 2.2. Fuzzy Mathematical Model and Solution Approach

The following fuzzy model is used to represent the SBRP, and it was previously proposed in [20]. This model aims to minimize the total distance traveled by the bus fleet, complying with the restrictions of bus capacity and the fuzzy students' maximum walking distance.

#### 2.2.1. Input Variables

 
*c*: Capacity of each bus.
 
*B*,  *b*: Set and index of buses, *b*=1,…, |*B*|.
 
*P*,  *p*: Set and index of possible stops, *p*=0,…, |*P*|, where *p*=0 indicates the school.
 
*E*,  *e*: Set and index of students, *e*=1,…, |*E*|.
 
*d*_*e*_: Maximum walking distance for student *e*.
 
*V*^*p*^: A set of vectors with pairs of coordinates representing the possible stops.
 
*V*^*e*^: A set of vectors with pairs of coordinates representing each student's home
 
*H*_*e*_: Maximum admissible tolerance for the distance that the student *e* could walk.
 
*T*: Maximum allowed tolerance to overload the bus capacity.

#### 2.2.2. Auxiliary Parameters

*D*: A distance function that indicates the cost between a pair of stops or between a student and a bus stop.
*C*_*pq*_^*P*^: A distance matrix between each pair of stops (*p*, *q*).
*C*_*ep*_^*E*^: A distance matrix between each pair of student-stop (*e*, *p*).
*c* ^*p*^_*i*_: Coordinates of the stop located on the *i* index of *V*^*p*^.
*c* ^*e*^_*j*_: Coordinates of the student located on the *j* index of *V*^*e*^.
  (1) $$\begin{array}{c} C_{pq}^{P}=D\left(c_{p}^{p},c_{p}^{p}\right),p\neq q0,p=q\;\forall p,\;\forall q\;\in P,\\ C_{ep}^{E}=D\left(c_{e}^{e}\;,c_{p}^{p}\right)e\in E,p\in P-\left\{0\right\}. \end{array}$$
  (2) $$\begin{array}{c} C_{pq}^{P}=D\left(c_{p}^{p},c_{p}^{p}\right),p\neq q0,p=q\;\forall p,\;\forall q\;\in P,\\ C_{ep}^{E}=D\left(c_{e}^{e}\;,c_{p}^{p}\right)e\in E,p\in P-\left\{0\right\}. \end{array}$$

#### 2.2.3. Decision Variables

 
*R*_*bm*_: Indicates the stop that is visited by bus *b* in the order *m*. |*R*_*bm*_| indicates the number of bus stops visited by the bus *b*.
 
*Z*_*e*_: Indicates the stop where the student *e* is picked up

#### 2.2.4. Objective Function

(3) $$\begin{array}{c} \text{Min}\overset{\left|B\right|}{\underset{b=1}{\sum }}\left(C_{0\left[1\right]}^{P}+\overset{\left|P\right|-1}{\underset{m=1}{\sum }}C_{\left[R_{bm}\right]\left[R_{bm+1}\right]}^{P}+C_{\left[\left|R_{bm}\right|\right]0}^{P}\right). \end{array}$$

#### 2.2.5. Constraints

≤^*f*^: A fuzzy comparison operator, indicating the fuzzy imprecision of the constraint (5).

(4) $$\begin{array}{c} \left\{R_{bm}|R_{bm}=p\right\}|\leq 1,\;\forall p\in P-\left\{0\right\},\forall b\in B,\forall m\in \left\{1,\ldots ,\left|P\right|\right\}, \end{array}$$

(5) $$\begin{array}{c} \left\{Z_{e}=p\right\}\subseteq \left\{C_{ep}^{E}\leq^{f}d_{e}\right\},\;\forall e\in E,\forall p\in P, \end{array}$$

(6) $$\begin{array}{c} \left|\left\{\exists m\;R_{bm}=Z_{e}\right\}\right|\leq cm\in \left\{0,\ldots ,\left|P\right|\right\},\;\forall b\in B,\forall e\in E, \end{array}$$

(7) $$\begin{array}{c} \left|\left\{R_{bm}=Z_{e}\right\}\right|=1,\;\forall e\in E. \end{array}$$

The objective function, equation (3), minimizes the total distance traveled by the entire bus fleet. Equations (4)–(7) represent the constraints needed for the solution to be achievable. Equation (4) guarantees that each stop is visited at most once, except for the final destination of all buses (school, *p* = 0). Equation (5) ensures that each person can reach their assigned bus stop. Equation (6) considers that the number of persons assigned to one route does not exceed the size of the bus. Finally, with equation (7), it is guaranteed that one bus visits each stop to which at least one person is allocated. Equation (8) is used to replace equation (5), and the following explanation argues why.

(8) $$\begin{array}{c} \left\{Z_{e}=p\right\}\subseteq \left\{C_{ep}^{E}\leq d_{e}+H_{e}\left(1-\alpha_{w}\right)\right\}. \end{array}$$

As can be seen in the previous model, equation (5) is where the model becomes a fuzzy model. The way of posing these constraints implies that the feasibility of a student reaching a stop becomes fuzzy (i.e., not crisp), and therefore it is possible to satisfy this constraint with different degrees of membership.

Let us see an example. If the maximum walking distance for students is 350 meters, then a student at a distance of 200 meters to a bus stop satisfies it with a grade of 1. On the other hand, if a student walks 355 meters, the degree of satisfaction with this constraint may be less than 1, but it may be higher than the membership if the stop is 370 meters away. On the contrary, if a student needs to walk 600 meters to reach a bus stop, then this stop may be considered unreachable with a grade of 0.

All these values (e.g., 355 or 600, for the walking distance) will depend on the admissible conditions and the allowed tolerance. These values will imply that solutions will have distinct degrees of compliance with the constraint. From the decision-making point of view, these relaxations may allow a modest increment in the distance the students need to walk to find a relaxed solution with a reduced cost of the objective function.

**Figure 1:**
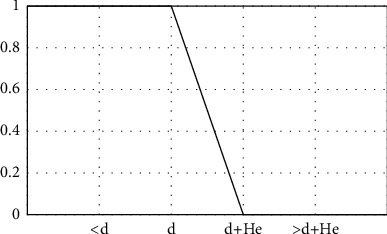
Membership function of the compliance of the fuzzy student's maximum walking distance.

To model this situation, it is necessary to define a tolerance *H*_*e*_, which determines the maximum admissible acceptance for a student's distance. If all the values of *H*_*e*_ are equal to 0, then the problem is reduced to the crisp case.  Figure 1 shows the function to measure the degree of compliance with the restriction of maximum walking distance, taking into account the distance *d* and the tolerance *H*_*e*_.

To comprehend this function, having *d* as the maximum walking distance and *H*_*e*_ as the maximum admissible tolerance, a bus stop distance less than or equal to *d* has a degree of compliance of 1. On the other hand, if the bus stop is located at a distance between *d* and *d* + *H*_*e*_, it has a degree of compliance in the interval [0, 1]. Finally, if the distance to reach the bus stop is greater than *d* + *H*_*e*_, the degree of compliance is 0, then it is assumed that the student *e* cannot reach this bus stop.

This is a linear function, and the parametric approximation method, based on the principles of parametric linear programming and the concept of alpha-cuts [15], can be applied. The alpha-cut notion applied to this case implies that different sets of feasible solutions are associated with each particular value of alpha, i.e., those solutions with a degree of feasibility (the accomplishment of the original conditions) greater than or equal to alpha. Consequently, with small values of alpha, a few relaxed solutions are considered feasible. Then equation (8) is used instead of equation (5).

With these changes, when *α*_*w*_=1, the problem remains crisp, and then students can only reach those bus stops settled at the original maximum walking distance, i.e., it is the most restrictive case. On the other hand, when *α*_*w*_=0, the students are allowed to reach those bus stops located at the original maximum distance plus the maximum tolerance, i.e., it is the greatest relaxation.

Authors who use the parametric approach to solve fuzzy problems usually use a uniform method to establish the aforementioned alpha-cuts, such is the case of [7, 9, 31]. On the other hand, in [16], an adaptive method is proposed where the alpha-cuts are calculated similarly to a binary search. This method offers certain advantages over the traditional uniform approach.

In general, selecting the alpha-cuts uniformly can bring with it various difficulties. Among these, the unnecessary exploration of some of (even of all) these alpha-cuts stands out when the maximum relaxation allowed does not contribute any differences to the quality of the solution. On the other hand, both in the traditional uniform way and the adaptive method, the set of crisp solutions obtained with each alpha-cut is not uniformly distributed in the space of the possible values of the objective function.

## 3. Conscious Exploration Strategy

The conscious exploration strategy for the obtention of alpha-cuts values takes into consideration a previous knowledge of the specific problem instance. In the case of the SBRP with fuzzy walking distance, the main feature to be taken into account is the distance between the students and the bus stops. This distance is calculated using the locations of each student and each bus stop. These pieces of information are part of the input variables of the model. Then a matrix that contains all the pairs of student-bus stop distances is calculated and becomes part of the auxiliary variables in the model (equation (2)).

To obtain the alpha-cuts, the mentioned distance values are split into three groups: (a) the distances that are less than or equal to *d*, (b) the distances between *d* and *d* + *H*_*e*_, and finally, (c) the distances that are greater than *d* + *H*_*e*_. Knowing that the distance in group (a) will always satisfy the constraint and distances in group (c) will always be beyond the allowed limits, then only the distances in group (b) are of value for the analysis. In this way, in an instance where all the distances are in groups (a) or (c), the relaxation that is given by *H*_*e*_ does not contribute to any improvement to the solution of the crisp instance. Thus, there is no need to explore any alpha-cuts, and the fuzzy solution will always be the same as the initial crisp solution.

**Figure 2:**
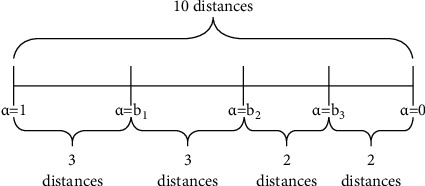
Graphic representation of the selection of alpha-cuts following the conscious strategy in an instance with 10 distances of interest.

The next step is to split group (b) as uniformly as possible into several subgroups that satisfy the desired quantity of alpha-cuts. For example, if an instance has ten distances in group (b), and we want to explore five alpha-cuts including 1 and 0, then four subgroups can be formed consecutively: the first three distances in the first subgroup, the following three in the second subgroup, the next two distances in the third subgroup, and the remaining two distances in the fourth subgroup. Now, with the last distance of each subgroup, except the last one, the alpha-cuts can be calculated, and three different alpha-cuts are obtained in addition to 1 and 0, which include the initial crisp problem and the maximum allowed relaxation problem. Then [1, b1, b2, b3, 0] represent all the alpha-cuts to be used in the parametric approach. It is to be noted that the alpha-cuts are set according to the characteristics of each instance. In Figure 2, a graphic representation of this example can be observed.

The complete method is described below.

### 3.1. Initialization

- Set *n* as the number of crisp subproblems to solve.
- Define *A* = {} as the array of alpha-cuts.
- Calculate the distance matrix (*M*) between each student-bus stop pair.

### 3.2. Obtaining Alpha-Cuts

(a) Make *A*_(0)_ = 1 as the crisp initial problem.
(b) Split the distances in the obtained matrix into three groups: (a), (b), and (c).
(c) If the group (b) is empty, go to step 3 with *A* = {1}.
(d) Sort the distances in group (b) and split this group into *n* − 2 subgroups.
(e) For each subgroup, calculate the alpha-cut (*α*) as follows:
  (9) $$\begin{array}{c} \left(\text{i}\right)\;\alpha =1-\frac{M_{\left(bi\right)}-d}{H}. \end{array}$$
Let *M*_(*bi*)_ be the last distance in the subgroup (i), *d* the maximum original walking distance, and *H* as the maximum allowed tolerance
(f) Add *α* to *A*.
(g) If the group (b) has more than *n* − 2 elements, make *A*_(*n*)_ = 0 as the maximum allowed relaxation problem. Otherwise, go to step 3 without the unnecessary relaxation of *α* = 0, since relaxation *A*_(*n* − 2)_ will yield the last solution of interest.

### 3.3. Solve

(a) Obtain a solution for each crisp instance corresponding to each value in *A* to get the fuzzy solution. Following the idea of [16], it is only needed to explore the central alpha-cut if the nonrelaxed crisp problem (*A*_0_) and the full relaxed crisp problem (*A*_*n*_) are different.

Equation (9) is the result of simplifying equation (7) in the previously exposed model.

## 4. Results and Discussion

To demonstrate the proposal's validity, a set of instances is obtained from the literature. In this case, 33 SBRP crisp instances from [32] were used and transformed into fuzzy instances with an H-tolerance of 20% of the maximum student's walking distance.

The characteristics of the instances can be appreciated in Table 1. The Instance column shows the identifier for each instance. Columns Students, Stops, and MWD show the number of students, the number of bus stops, and the maximum students' walking distances, respectively. All these columns correspond to original instances from [32]. The Variation column shows the number of new student-stop pairs that arise after applying the tolerance (20%). These student-stop pairs are the ones that could bring about changes in fuzzy solutions. Finally, the Group column shows a group identifier to organize the analysis of the results conducted in the next section.

## 5. Results

In the experimentation, three approaches for obtaining the necessary alpha-cuts were used. The proposed approach, named Conscious, the Adaptive approach presented by [16], and a uniform approach previously used in [20], with *α* = {1, 0.75, 0.5, 0.25, 0}.

The number of crisp instances to be solved corresponds to the number of generated alpha-cuts by applying the parametric approach to solve these fuzzy instances. A metaheuristics-based method reported in [33] was used to find the solution for each crisp instance.

The cost (integer part) of the obtained results can be seen in Table 2. In this table, the Id column represents the identifier of the instance. Column *α* = 1 shows the result of the original crisp instance (the same value for all strategies). The following twelve columns show the results of the objective function obtained with each strategy for the selected alpha-cuts (Conscious, Adaptive, and Uniform). The last columns with values on each strategy represent the solutions with maximum relaxation (*α* = 0). Cells with “—” represent crisp instances that do not need to be solved.

We divide the instances into three subgroups (see the Group column in Table 1).

In the case of the first subgroup (with 10 out of 33 instances, i.e., 30%), the first ten instances of Table 2 are those instances in which the maximum relaxation of 20% does not generate new student-bus stop pairs. So, it is identified that it is unnecessary to solve these instances for any relaxation of the original problem. As shown in Table 2, the Conscious strategy executes only one crisp problem, the nonrelaxed one. In the case of the Adaptive method, two solutions were found: for the nonrelaxed problem and the full-relaxed problem. Finally, the Uniform strategy found an identical solution for each preestablished relaxation level. In this case, the resolution of the intermediate relaxations could be avoided by applying a principle similar to the rest of the strategies, solving and comparing the nonrelaxed problem and the full-relaxed problem at the beginning.

The results of this first group let us confirm that the Conscious strategy allows for time savings in solving the fuzzy problem. This time-saving comes in since it avoids executing the solution method for relaxations that do not bring any change to the original problem and, thus, the best (thus, the only) solution is that of the initial problem, i.e., the nonrelaxed one. In general, the Conscious method allows for savings of at least 50% of the computational cost concerning the other methods in 30.3% of the instances used in the experiments.

The second subgroup (with 7 out of 33 instances, i.e., 21.2% of the instances) are those instances in which the solution mechanism cannot find distinct solutions for the different levels of relaxation. It is important to note the difference concerning the first subgroup. In the first subgroup, it is impossible to obtain an improvement based on the relaxation because no student can reach additional stops, while in this second subgroup, the relaxation allows to analyze more options, but this does not allow to get better solutions. These instances are in the next seven rows in Table 2. In these instances, the Conscious and the Adaptive strategies only evaluated the nonrelaxed and full-relaxed instances. The Uniform strategy considers all the preestablished alpha-cuts offering the same solution. In general, the Conscious and Adaptive methods allow for savings of 80% of the computational cost concerning the Uniform method in 20% of the instances of the experiments.

The third subgroup contains the last sixteen instances of Table 2 (16 out of 33 instances, i.e., 48.5% of the instances). Different objective function values were found in these instances for distinct alpha-cuts on each strategy. Using this group, we present four criteria that show the superiority of the Conscious technique over the other methods.

The first criterion is that, on average, the number of different trade-off solutions (solutions with different values of the objective functions) in each fuzzy instance when using the Conscious strategy is 3.938, slightly higher than the 3.875 obtained with the Adaptive method and than the 3.563 achieved with the Uniform strategy (see Table 3). This aspect allows us to confirm that, with the Conscious technique, more diverse results are found. For instance, in instance 48, the Conscious method produces four different solutions (with cost 60, 56, 52, and 43), the Adaptive method also produces four different solutions (with cost 60, 51, 49, and 43), while the Uniform method produces three different solutions (with cost 60, 49 and 43). As the extreme values are identical, the Conscious and Adaptive strategies produce two new interesting, relaxed solutions while the Uniform method only produces one.

The rest of the criteria are based on analyzing the differences in the cost of the objective function between two consecutive relaxations. For each fuzzy solution of an instance where five alpha-cuts were studied, there are four distances between the costs of two adjacent relaxations. The ideal situation is that the difference in terms of cost between them is similar since this implies a similar difference in the quality of the solutions. This situation is more interesting for a decision-maker because it implies having options with different costs to evaluate different trade-offs. For example, in an instance where the cost of the original crisp solution is 100 and the cost of the more relaxed solution is 60 for a decision-maker, an ideal set of intermediate relaxations are those with costs of 90, 80, and 70 because they are equally separated in terms of costs (all at equal distance of 10). This is more interesting for a decision-maker than intermediate relaxations with costs of 97, 95, and 92. In this last case, the distances between each successive relaxation are 3, 2, 3, and 32 (e.g., 32 is the distance between 92 and 60).

To generalize this analysis, it is convenient to normalize the distances to eliminate the noise caused by the different sizes of the instances. So, the nonrelaxed solution corresponds to 1, while the full-relaxed solution corresponds to 0. This allows analyzing all instances from a uniform point of view. Following the previous example, according to this normalization, the ideal case with four distances of 10 in the interval [60, 90] corresponds to a normalized distance of 0.25. On the other hand, the normalized values of distances 3, 2, 3, and 32 in the same example are 0.075, 0.05, 0.075, and 0.8.

The normalization has the following steps:

1. Find the differences between the solutions based on alpha-cuts concerning the previous one, with less relaxation.
2. Find the difference between the nonrelaxed and the full-relaxed solution.
3. Find the ratio between each difference computed in step 1 and the one calculated in step 2.

Table 3 shows the data obtained after the normalization for all the instances. In this table, the first row corresponds to the first criterion already described, showing the average number of different solutions obtained by each strategy. Rows 2, 3, and 4 present the number of normalized values in each indicated interval obtained by each technique. Finally, the fifth row shows the standard deviation of the normalized values for each strategy.

The second criterion to be considered in the analysis is that most differences should be as close as possible to the ideal value of 0.25. For the third criterion, the standard deviation of the normalized values is considered, with a lower deviation being an indicator of higher quality.

Table 3 shows how the Conscious strategy contains more normalized values in [0.1, 0.4], in [0.15, 0.35], and in [0.2, 0.3] than the other techniques. This difference is accentuated in the narrowest interval. Likewise, the standard deviation presented by the normalized values in the Conscious strategy is slightly lower than that of the Adaptive and Uniform methods. These criteria demonstrate that the Conscious technique provides better levels of quality in the trade-off values to be taken into account by decision-makers.

**Figure 3:**
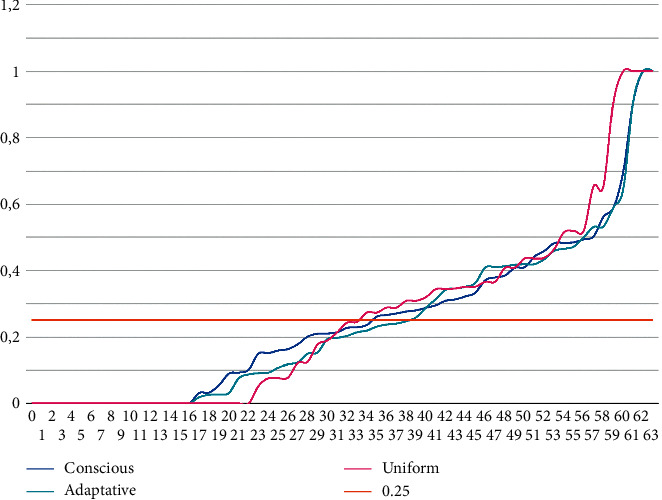
Normalized values of distances in terms of the cost of two consecutive relaxations in all instances. The dotted grey line represents the ideal value (0.25).

Finally, Figure 3 presents the fourth criterion. It shows the 64 normalized values (4 distances, 16 instances) of all instances for each strategy analyzed. The values are in ascending order. The plot displays how these values come close to the desired value (0.25) earlier with the Conscious strategy and move away from this ideal later than with the rest of the strategies. Likewise, the Adaptive method shows better performance in this aspect than the Uniform method.

## 6. Conclusions

This paper proposes a strategy that makes use of the characteristics of the instances of the School Bus Routing Problem to focus the search towards interesting relaxations in the solutions of the fuzzy model with fuzzy students' maximum walking distance.

This proposed Conscious strategy is advantageous concerning the traditional way of obtaining the possible relaxation values (Uniform method) in all the instances used. It also presents advantages concerning the Adaptive method in terms of the amount and quality of the different solutions found and computational cost savings by avoiding relaxations on instances where these do not provide new solutions.

Despite the advantages of the Conscious method, it is worth noting that the Adaptive method has general characteristics that allow it to be used in multiple combinatorial optimization problems. On the other hand, the proposed method must be adapted according to the characteristics of each particular problem.

In future work, we recommend using the Conscious strategy in other combinatorial optimization problems with fuzzy elements, such as the Maximum Coverage Location Problem with fuzzy coverages. Also, it is motivating for future work to compare the presented results with a multiobjective approach that minimizes the fuzzy constraint violation and the total distance traveled.

## Figures and Tables

**Table 1:** Characteristics of the instances to analyze.

Instance	Students	Stops	MWD	Variation	Group
1	25	5	5	0	1
2	25	5	5	0	1
4	25	5	10	0	1
5	25	5	20	8	3
6	25	5	20	3	3
9	50	5	5	0	1
10	50	5	5	0	1
12	50	5	10	0	1
17	100	5	5	0	1
18	100	5	5	0	1
19	100	5	10	8	3
24	100	5	40	33	3
27	50	10	10	13	3
30	50	10	20	42	2
39	100	10	40	56	2
41	200	10	5	0	1
42	200	10	5	0	1
44	200	10	10	32	2
45	200	10	20	142	3
47	200	10	40	124	2
48	200	10	40	151	3
56	100	20	40	141	3
58	200	20	5	15	2
64	200	20	40	306	3
72	400	20	40	635	3
87	400	40	40	1316	3
88	400	40	40	1113	3
95	800	40	40	2593	3
96	800	40	40	2416	3
103	400	80	40	2495	3
105	800	80	5	262	2
111	800	80	40	4381	3
112	800	80	40	4511	3

**Table 2:** Results obtained after applying each of the three methods, grouped by the group id of the instance.

Id	*α* = 1	Conscious				Adaptive				Uniform			
		*α*_1_	*α*_2_	*α*_3_	0	*α*_1_	*α*_2_	*α*_3_	0	0.75	0.5	0.25	0
1	141	—	—	—	—	—	—	—	141	141	141	141	141
2	162	—	—	—	—	—	—	—	162	162	162	162	162
4	196	—	—	—	—	—	—	—	196	196	196	196	196
9	239	—	—	—	—	—	—	—	239	239	239	239	239
10	197	—	—	—	—	—	—	—	197	197	197	197	197
12	216	—	—	—	—	—	—	—	216	216	216	216	216
17	311	—	—	—	—	—	—	—	311	311	311	311	311
18	259	—	—	—	—	—	—	—	259	259	259	259	259
41	668	—	—	—	—	—	—	—	668	668	668	668	668
42	428	—	—	—	—	—	—	—	428	428	428	428	428

19	230	—	—	—	230	—	—	—	230	230	230	230	230
24	12	—	—	—	12	—	—	—	12	12	12	12	12
27	144	—	—	—	144	—	—	—	144	144	144	144	144
44	410	—	—	—	410	—	—	—	410	410	410	410	410
47	103	—	—	—	103	—	—	—	103	103	103	103	103
58	476	—	—	—	476	—	—	—	476	476	476	476	476
105	2582	—	—	—	2582	—	—	—	2582	2582	2582	2582	2582

5	112	112	112	110	97	110	110	97	97	112	110	97	97
6	103	103	103	92	92	103	103	92	92	103	103	103	92
30	122	115	105	105	92	121	121	105	92	109	105	102	92
39	61	53	53	41	41	53	41	41	41	41	41	41	41
45	339	330	330	330	330	339	330	330	330	339	330	330	330
48	60	56	52	43	43	60	51	49	43	49	49	43	43
56	21	16	16	11	4	16	8	8	4	16	16	8	4
64	54	54	50	33	29	54	49	33	29	54	40	30	29
72	95	86	68	68	63	93	79	63	63	79	63	63	63
87	217	216	206	203	190	216	205	202	190	210	202	202	190
88	78	77	70	62	53	76	70	64	53	76	66	53	53
95	415	405	405	404	393	405	402	393	393	415	415	393	393
96	220	213	204	184	177	214	196	195	177	204	195	188	177
103	141	133	129	123	110	130	129	123	110	130	129	123	110
111	324	315	309	294	294	318	313	305	294	315	297	294	294
112	139	131	122	112	104	135	127	115	104	127	115	110	104

**Table 3:** Criteria to take into account for the analysis.

Criteria	Conscious	Adaptive	Uniform
Average of different values	3.938	3.875	3.563
Values in [0.2, 0.3]	14	9	7
Values in [0.15, 0.35]	22	15	15
Values in [0.1, 0.4]	27	21	21
Standard deviation	0.243	0.246	0.285

## Data Availability

The data used to support the findings of this paper are available at https://drive.google.com/drive/folders/1azYEsY2Xs0vYdQ5aLgF-kvR_Do3xv_z1?usp=sharing.
